# Scaling the collaborative care model in obstetrics: Multilevel determinants to guide implementation

**DOI:** 10.1002/pmf2.70356

**Published:** 2026-07-07

**Authors:** Alexandra Turco, Andrea Chu, Emily Feinberg, L. G. Ward, A. Rani Elwy, Emily S. Miller

**Affiliations:** ^1^ Warren Alpert School of Medicine at Brown University Providence Rhode Island USA; ^2^ Hassenfeld Institute for Child Health Innovation Brown University School of Public Health Providence Rhode Island USA; ^3^ Women and Infants Hospital Providence Rhode Island USA; ^4^ Center for Behavioral and Preventative Medicine Brown University Health Providence Rhode Island USA; ^5^ Center for Health Optimization and Implementation Research VA Bedford Healthcare System Bedford Massachusetts USA; ^6^ Department of Psychiatry and Human Behavior Warren Alpert School of Medicine Providence Rhode Island USA

**Keywords:** collaborative care, health disparities, maternal mental health, mental health services, perinatal mood and anxiety disorders, postpartum depression

## Abstract

**Objectives:**

The Collaborative Care Model (CCM) is an evidence‐based approach to care that improves health outcomes in primary care settings by integrating mental health into routine medical care. While recent adaptations have tailored the CCM for use in obstetric settings to address perinatal mental health (PMH) concerns, real‐world implementation remains limited. Bridging this gap between research and practice requires an understanding of contextual factors influencing adoption and sustainment. As a sub‐study of the Collaborative Care Model for Perinatal Wellness Support Services—Population‐Level Upstream Systems Change (COMPASS+) Randomized Trial, our objective was to identify key factors modifying the implementation of the perinatal CCM (pCCM).

**Study design:**

We conducted a qualitative implementation study guided by the Exploration, Preparation, Implementation, Sustainment (EPIS) framework to evaluate facilitators and barriers of pCCM implementation during the Preparation phase of pre‐implementation. Using purposive sampling, we recruited clinical and administrative key informants from five obstetric clinics and one birthing hospital, all affiliated with COMPASS+ Trial. Semi‐structured interviews were designed to elicit determinants at multiple contextual levels. Data were analyzed using the Rapid Qualitative Analysis process. Coding and thematic analysis were guided by the EPIS domains—inner context, outer context, bridging factors, and innovation characteristics.

**Results:**

A total of 20 individuals were interviewed prior to thematic saturation. Determinants influencing adoption and sustainability of the pCCM were mapped across EPIS domains. Several cross‐cutting barriers emerged on the clinic level related to limited office space and workflow changes to accommodate the pCCM, at the population level including societal stigma regarding the use of psychotropic medications during pregnancy, and among bridging factors such as need for mental health referral networks. Participants also identified multiple facilitators that support integration of the CCM into obstetric settings, such as increased medical touchpoints during pregnancy, staff and leadership buy‐in, and preexisting comfort with obstetric clinicians. The need for site‐level adaptation was an underlying theme, underscoring the opportunity to tailor implementation strategies to local contexts.

**Conclusion:**

The CCM is well suited to obstetric care but requires context‐specific adaptation to support adoption and sustainability. Implementation strategies must address multilevel barriers and leverage existing facilitators to bridge the gap between research and practice.

## 1 INTRODUCTION

Perinatal mood and anxiety disorders (perinatal mental health condition) are among the leading causes of maternal mortality in the United States [1, 2]. An estimated 600,000–900,000 people are affected by perinatal mental health conditions annually in the United States [3], with wide‐reaching health impacts on birthing people, the parent dyad, and child health.

Untreated perinatal mental health conditions can lead to safety concerns including suicide and substance use, accounting for 23% of pregnancy‐related deaths [4]. Further, perinatal mental health conditions affect quality of life by increasing risk of relationship and occupational troubles [5, 6]. These conditions also negatively affect infant outcomes by contributing to lower breastfeeding rates and alterations in parent‐child interaction [7, 8, 9, 10, 11]. Effects of untreated PMADs on children last well past infancy, extending into adulthood, and include decreased school performance, insecure attachment styles, and poor psychological and behavioral outcomes [12, 13, 14].

Despite the significant disease burden, perinatal mental health conditions often go undiagnosed and undertreated; 50%–70% of people with a perinatal mental health condition remain undiagnosed [15, 16]. This gap in diagnosis is multifactorial, reflecting inconsistent screening, lack of meaningful patient engagement with screening materials, and limited clinical evaluation. Even with an accurate diagnosis, less than 30% of birthing people receive treatment, despite available, effective therapies [3].

Because current delivery of care for perinatal mental health conditions is inadequate, systems‐based solutions, such as the Collaborative Care Model (CCM), have been suggested. The CCM has been proven as an effective strategy to embed mental health care within existing medical care structures, expanding access and quality in over 80 randomized controlled trials [17, 18]. The model encompasses three unique components of care: (1) a Care Manager (CM), (2) a patient registry, and (3) interdisciplinary care meetings. [18, 19, 20] Specifically, within a perinatal CCM (pCCM), the CM functions at the center of a birthing person's care, responsible for treatment plan creation, delivery of psychotherapy, provision of supportive check‐ins, and care coordination. A centralized source for perinatal mental health care, the CM has knowledge of community resources, including a referral network for needs that extend beyond the pCCM programming. The CM utilizes a patient registry to systematically monitor response to treatment and to oversee population‐level health metrics. Weekly interdisciplinary meetings between the CM and a supervising psychiatrist allow for discussion of new people referred to the program, as well as those who are not improving, to guide stepped care.

The CCM has robust evidence of efficacy in primary care [21, 22, 23]. Emerging evidence supports its adaptation and implementation in perinatal settings [24, 25]. Despite its promise for improving perinatal mental health outcomes, real‐world applications of the CCM in obstetrics are limited. Structural and contextual differences exist between primary care and obstetric care settings, necessitating tailored adaptations and implementation strategies to support CCM delivery [26, 27, 28, 29].

The Collaborative Care Model for Perinatal Wellness Support Services—Population‐Level Equity‐Centered Systems Change (herein referred to as COMPASS**+**) Trial is a large‐scale, pragmatic, cluster‐randomized trial designed to evaluate the effectiveness of a community co‐designed pCCM and to assess implementation strategies supporting its delivery [30]. The aim of this sub‐study, conducted during the pre‐implementation phase, was to qualitatively examine determinants influencing organizational readiness for the pCCM to guide implementation planning.

By exploring existing barriers to perinatal mental health care, clinician and staff attitudes toward integrated behavioral health, and patient‐level factors influencing engagement, the COMPASS+ team sought to identify contextual determinants anticipated to influence implementation of a pCCM. This evaluation was designed to inform implementation planning, including refinement of clinic workflows, identification of staffing needs and care manager competencies, development of training approaches, and adaptation of referral and communication processes to fit individual clinic environments. In addition, this study was designed to include a broad range of clinical roles across diverse obstetric practice settings, allowing the findings to generate practical insights that may inform implementation efforts in other perinatal clinics considering pCCM adoption. Specifically, the results may help other programs anticipate common barriers and facilitators, tailor implementation strategies to local clinical contexts, and structure pre‐implementation stakeholder assessments intended to support readiness and uptake.

## 2 METHODS

### 2.1 Conceptual framework

The Exploration, Preparation, Implementation, Sustainment (EPIS) framework guided our investigation of barriers and facilitators to pCCM implementation. EPIS conceptualizes implementation as a dynamic four‐stage process [31]. The Exploration phase involves problem identification and stakeholder planning. Once a specific action is decided upon, the Preparation phase focuses on the identification of potential facilitators and barriers and the development of evidence‐based implementation strategies. The Implementation phase marks active rollout of the intervention and is characterized by ongoing monitoring, which extends into the Sustainment phase to support long‐term integration and maintenance.

**FIGURE 1:**
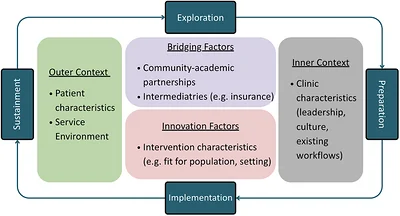
EPIS framework. EPIS, Exploration, Preparation, Implementation, Sustainment.

EPIS articulates interconnected domains that overlie all phases of implementation: the inner context, outer context, innovation factors, and bridging factors. When applied to a pCCM, the inner context denotes factors within the obstetric clinic, such as staffing, leadership, existing workflows, and culture. The outer context denotes population characteristics, societal beliefs regarding perinatal mental health, and relevant existing infrastructure. Innovation factors describe inherent intervention characteristics. Lastly, bridging factors are the interconnected entities tying the inner to the outer context, including community‐academic partnerships and intermediaries (e.g., insurance providers). Domains with specific applications to pCCMs are described in Figure 1.

### 2.2 Data collection

We conducted a developmental evaluation to better understand determinants of current perinatal mental health care practices, barriers and facilitators to practice change, and the feasibility of implementing a pCCM [32]. Our research occurred during the Preparation (pre‐implementation) phase of COMPASS+ using semi‐structured interviews with stakeholders from six sites: five obstetrics clinics participating in the pCCM and one affiliated birthing hospital (see the Interview Guide in the Supporting Information). In accordance with COMPASS+ Trial location, all data collection occurred within the state of Rhode Island.

**FIGURE 2:**
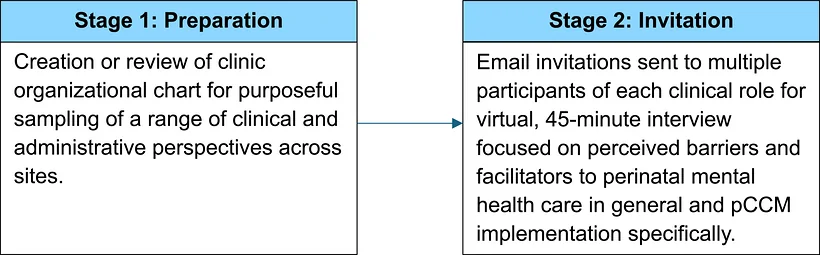
Recruitment. pCCM, perinatal Collaborative Care Model.

Recruitment comprised two stages, described in Figure 2. There was no maximum number at each clinic that could participate; anyone interested in participating was included in the study.

All interviews were conducted by the first author, who had no prior professional or personal relationships with interviewees. Prior to data collection, the interviewer received training in qualitative interviewing methods from an experienced qualitative researcher on the COMPASS+ team. Interviews were recorded and transcribed via Zoom software and manually reviewed by the interviewer for accuracy. Interviews were conducted until thematic saturation was achieved, as defined by Hennink et al. [33]. Saturation was determined during preliminary data analysis when no new major themes relative to study objectives emerged upon structured memo creation. In our study, this occurred after 20 interviews.

### 2.3 Data analysis

**FIGURE 3:**
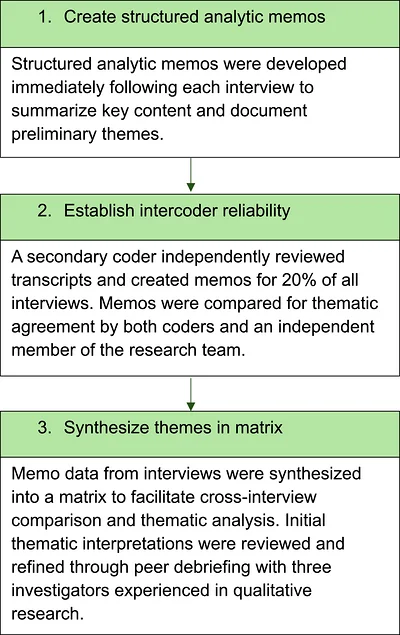
Data analysis.

The four EPIS domains guided deductive thematic analysis of barriers and facilitators, completed using the Rapid Qualitative Analysis Framework [34, 35] comprising three main stages, as described in Figure 3.

## 3 RESULTS

A total of 20 participants were interviewed, at which point thematic saturation was achieved. Participant characteristics are summarized in Table 1.

**TABLE 1:** Number and type of participant interviewed.

Role of participant	Location	Number interviewed
Obstetrician/Gynecologist (OB/Gyn)	Academic clinic	3
	Community clinic	3
Midwife	Academic clinic	1
	Community clinic	2
Social worker	Academic clinic	1
	Community clinic	2
Medical assistant	Academic clinic	1
	Community clinic	1
Practice manager	Academic clinic	1
	Community clinic	1
Perinatal psychiatrist	Birthing hospital	2
Psychologist	Academic clinic	1
	Community clinic	1

**FIGURE 4:**
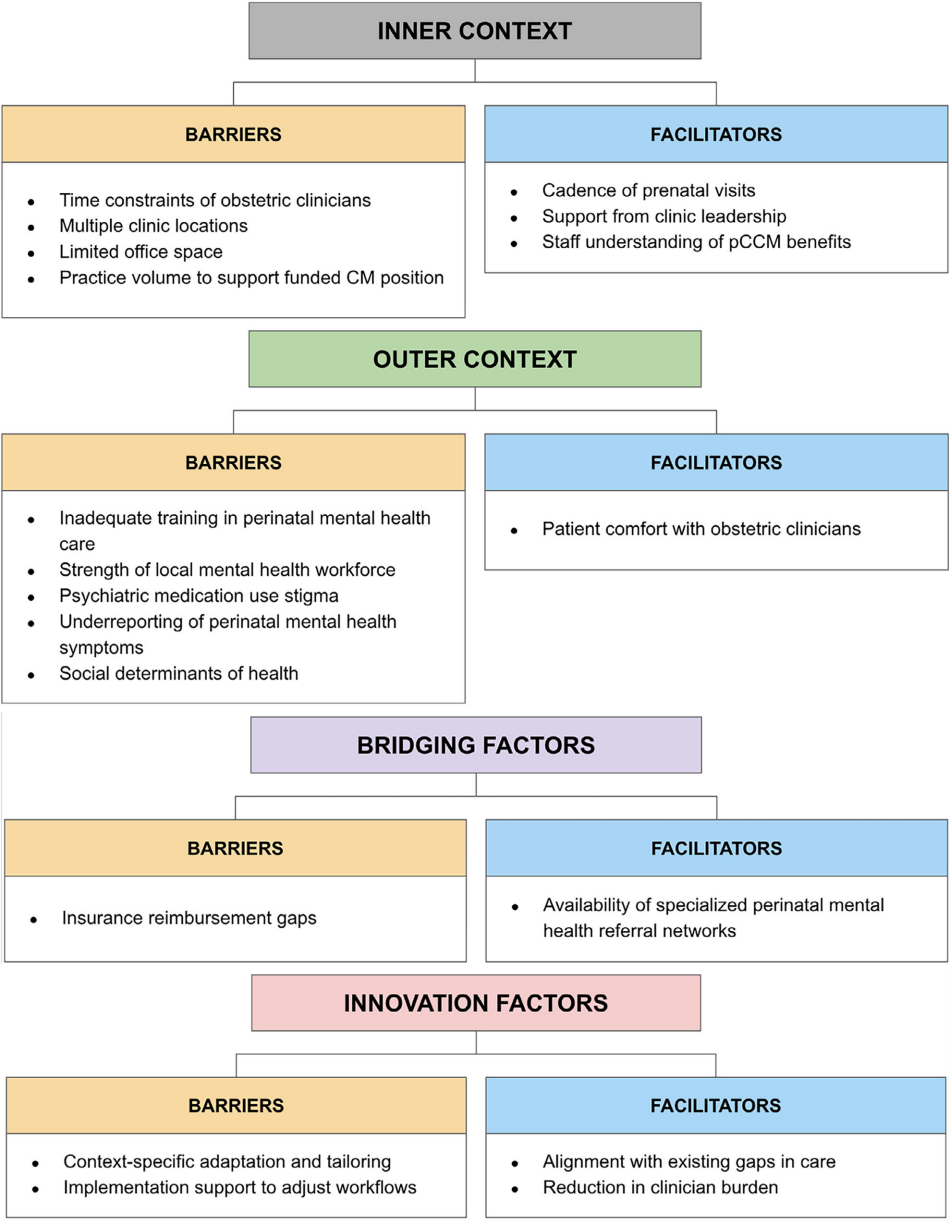
Thematic results by EPIS domain. EPIS, Exploration, Preparation, Implementation, Sustainment; pCCM, perinatal Collaborative Care Model.

Key themes identified are presented by EPIS domains below in Figure 4 and explored with quotations in Table 2.

**TABLE 2:** Determinant with exemplary quote.

EPIS domain	Determinant	Exemplary quote(s)
*Inner context*		
Barrier	Time constraints of obstetric clinicians	“A 15 min prenatal visit to treat a pregnancy, and then also treat a psychological concern, to fully assess that, and then make a medication adjustment, I think is a big ask."—OB/GYN
	Multiple clinic locations	“If I could be doing an actual warm handoff, that would be great. I don't know how realistic that is for a place that has multiple office locations.”—Midwife
	Limited office space	“I think space at {our clinic} is a challenge. Even with social work, they usually come and meet with the patients in the room. But then we need the exam rooms for the next patient.”—LICSW
	Practice volume to support funded CM position	“Having a Care Manager would be great, but, if I'm the only one in the office, and I've got 3 prenatal visits in my day, that's not enough to support a Care Manager.”—OB/GYN
Facilitator	Cadence of prenatal visits	“I think it's easier during pregnancy, because you're seeing people at very regular intervals.”—Practice manager
	Support from clinic leadership	“If the provider really believes in the model, they're going to be more persuasive about the benefits."—Medical Assistant
	Staff understanding of pCCM benefits	“Some staff will naturally understand the value pretty quickly, and others are a little bit skeptical… It's about finding ways to effectively communicate and demonstrate the benefits of the program both to the providers and to the patients.”—OB/GYN
*Outer context*		
Barrier	Inadequate training in perinatal mental health care	“The familiarity with medications, side effects, and risk {is a barrier}. There's a couple that I've used over the years, but if they don't respond to that, then I don't feel comfortable going further, adding a medication, or keep on going up on the dose.”—OB/GYN
	Strength of local mental health care workforce	“Even if they make that phone call, a lot of providers are fully booked up, or they're booked out for months.”—Midwife
	Psychiatric medication use stigma	“There is this cultural component of prioritizing a fetus over the health of a pregnant person, especially their mental health. The second that people become pregnant, you sacrifice for your baby: you sacrifice what you're eating, you sacrifice what you're doing, and you sacrifice medication that you're taking at certain expenses.”—Midwife
	Underreporting of perinatal mental health symptoms	“Sometimes there is discomfort with disclosure of symptoms related to views of friends or family members of pregnancy, discouraging someone from talking about their symptoms.”—Perinatal psychiatrist “Unfortunately, some patients are from populations where there may be some lack of trust in the medical system, or fear of repercussions. If a pregnant woman or a mom is saying “I feel depressed”—They have this notion that maybe their babies may be removed from their care, that DCYF will get involved.”—Perinatal psychologist
	Social determinants of health	“You can have someone engaged in daily psychotherapy, but if they don't have stable housing, they're going to be chronically destabilized.”—LICSW
Facilitator	Patient comfort with obstetric clinicians	“It {pCCM} is appealing because people are usually more comfortable going to their OB/Gyn versus a psychiatry clinic. And it's appealing because psychiatry clinics take a long time to get in a lot of the time, and a lot of the time they have a higher Co‐Pay."—Medical Assistant
*Bridging factors*		
Barrier	Insurance reimbursement gaps	“In my opinion, success {of a pCCM} would mean creating a sustainable, billable model.”—OB/GYN
Facilitator	Availability of specialized perinatal mental health referral networks	“I have a referral network to send people to behavioral health that is specific for perinatal health. I feel so lucky that I have that as an option, because I know a lot of providers work in places where they don't have access to these resources.”—Midwife
*Innovation factors*		
Barrier	Context‐specific adaptation and tailoring	“As part of this part of this project…we're asked to increase screening rates…before the services are delivered, you're forcing clinics to uncover more problems than they may feel equipped to solve."—Practice manager
	Implementation support to adjust workflows	“Figuring out the boundaries of when we call social work versus when we reach out to a Care Manager…can be challenging.”—OB/GYN
Facilitator	Alignment with existing gaps in care	“Being able to find not only available but appropriate resources, and helping establish long term care, will make a significant difference in our community, not just for our patients.”—Medical Assistant
	Reduction in clinician burden	“Because obstetrics is extremely emotionally demanding on providers, a Care Manager could be one of the ways to help to lift that burden so that the providers are able to provide care and not feel emotionally burnt out by also trying to manage things that are outside of the scope of their practice.”—Perinatal psychologist

### 3.1 Inner context

#### 3.1.1 Inner context—Barriers

Participants endorsed multiple barriers at the visit, clinic, and practice levels. Clinicians most frequently cited time constraints as a major barrier to delivering perinatal mental health care. Routine visits for prenatal care last 15–20 min and must accommodate obstetric assessments, preventive care counseling, and delivery and postpartum planning. Within this time constraint, clinicians are being asked to screen for perinatal mental health conditions, evaluate reported symptoms, and, if applicable, establish a diagnosis. Many cited, despite understanding the clinical need for perinatal mental health care, concerns that conversations about mental health might be limited by the visit duration. These challenges are further amplified when interpreter services are required, given additional time demands for communication.

Clinic characteristics were identified as a potential barrier to pCCM implementation. Staff noted that some practices have multiple clinic locations, requiring a CM to travel for on‐site support. CM scheduling may therefore require site‐level implementation support with ongoing evaluation. Further, because the CM will be engaging in sensitive conversations, to prevent interruptions and avoid throughput challenges, participants preferred a private and reserved location apart from clinic exam rooms. Consequently, office space limitations were identified as a barrier to pCCM implementation.

Lastly, practice volume considerations were identified as a barrier to the expansion of pCCMs. Sustaining a CM position requires sufficient patient volume to justify a salaried position. Despite the high prevalence of perinatal mental health conditions, participants anticipated that lower patient volumes in smaller clinic settings could constrain the financial feasibility of maintaining a dedicated CM.

#### 3.1.2 Inner context—Facilitators

Participants noted that longitudinal monitoring of mental health symptoms with CM support would fit well given the frequent cadence of prenatal care visits. Because pregnant people are already expecting regular touchpoints with the medical system, the opportunity to centralize mental health services within obstetric clinics was clear.

Another facilitator of pCCM implementation is support from clinic leadership. Because pCCM implementation is limited, having a clear advocate for the model coming from a trusted staff member was identified as a strategy to encourage model adoption. Participants described more willingness to engage with the pCCM based on perceived support from clinic leadership.

Further, staff education on clinical evidence for the CCM, along with a thorough understanding of how the model will affect patient care, was identified as a key implementation strategy facilitating adoption and sustainability.

### 3.2 Outer context

#### 3.2.1 Outer context—Barriers

Within the outer context, barriers to pCCM implementation reflected broader limitations in medical education for obstetric clinicians. Clinicians described inadequate training in the diagnosis and treatment of perinatal mental health conditions, which limited their confidence and ability to fully engage as a member of the pCCM. Clinicians expressed particular hesitation regarding psychiatric medication management, with many citing difficulties titrating medication regimens to symptom remission or discomfort prescribing certain medication classes.

Participants also identified that a potential barrier to pCCM sustainability is the availability of external mental health resources. While the CM provides initial behavioral intervention, some individuals will require more intensive psychotherapy or specialty mental health services. The CM's ability to triage individuals to higher levels of care could be constrained by local or regional specialized mental health care workforce availability.

On the patient level, cultural barriers to perinatal mental health care were identified. Obstetric and psychology clinicians describe deeply ingrained beliefs among some patients and patients’ families that psychotropic medications are incompatible with pregnancy. As such, pregnant people on medications such as selective serotonergic reuptake inhibitors (SSRIs) often stop medication treatment altogether upon a positive pregnancy test.

Additionally, drawing on prior clinical experiences, several interviewees described challenges with underreporting of perinatal mental health symptoms. Deeply embedded societal narratives frame pregnancy as a time of unattenuated joy, reinforced by media, families, and even clinicians. Patients’ symptoms of perinatal mental health conditions can come into conflict with these social norms. As a result, pregnant and postpartum people may underreport symptoms or engage less fully with clinical evaluation.

Further complicating patient engagement in perinatal mental health care, participants expressed how some may withhold or minimize perinatal mental health symptoms due to fears of involvement from child protective agencies, especially among individuals with prior experiences in state or carceral systems.

Lastly, clinicians described social determinants of mental health as a barrier to successfully managing perinatal mental health conditions. Despite integrated and expanded behavioral health support, many interviewees feared remission of perinatal mental health conditions may be stalled by social determinants of mental health, such as housing, food security, and freedom from violence. Therefore, benefits of the CM would be most fully realized if clinics had additional services embedded to synergistically address health‐related social needs.

#### 3.2.2 Outer context—Facilitators

Participants described pCCM adoption being facilitated by existing trust between patients and obstetric clinicians. Frequently, pregnant people have established care at an obstetric clinic prior to pregnancy or have had prior experience interacting with obstetric clinicians. Therefore, centralizing mental health services at the obstetric clinic fosters a lower barrier to entry into mental health services compared to referral to a psychiatry clinic.

### 3.3 Bridging factors

#### 3.3.1 Bridging factors—Barriers

One key bridging factor that limits the expansion of pCCM is variability in insurance reimbursement. While collaborative care billing codes were formally integrated into the Current Procedural Terminology in 2018 [36], the availability of these codes and associated reimbursement varies among practice settings and payers. Further, CCM billing codes may not always be accepted outside of the primary care setting. As a result, close collaboration with experienced billing teams may be important to support appropriate coding and reimbursement for pCCM services.

#### 3.3.2 Bridging factors—Facilitators

The availability of academic‐community partnerships was cited by participants as a potential facilitator of pCCM success and care quality. Rhode Island, the setting of the COMPASS+ Trial, is a state unique for its perinatal mental health resources, including the nation's first partial hospital program for perinatal mental health conditions. Clinicians cited these resources as tailwinds for pCCM implementation.

### 3.4 Innovation factors

#### 3.4.1 Innovation factors—Barriers

Regarding the pCCM innovation itself, participants cited the main barrier as the need to modify existing clinical workflows, including screening protocols and referral pathways. To reliably identify individuals who would benefit from integrated behavioral health services, screening processes specific to the clinic's workflow must be optimized. Participants cited challenges and opportunities for each clinic to determine workflows that support mental health care provision without disrupting clinical throughput.

Further, integration of a CM necessitates deliberate revision of clinic procedures to support patient privacy and comfort while preserving obstetric care efficiency, representing a theme coupling the inner context to intervention domains. Identification of perinatal mental health concerns should prompt connection to pCCM services, ideally through a “warm handoff,” in which the obstetric clinician introduces the patient in real‐time to the CM. However, time constraints on obstetric clinicians, identified as an Inner Context barrier, coupled with competing clinical demands of the CM necessitate pre‐implementation planning and clearly delineated workflows. Additionally, clinics with existing social work services will also need to coordinate roles to clarify the scope of the CMs to prevent duplication of services.

#### 3.4.2 Innovation factors—Facilitators

Despite the need for tailored implementation support, participants identified a clear need for the services provided within a pCCM. Participants expressed how the integration of behavioral health services within obstetric clinics addresses a clear bottleneck in the perinatal mental health care cascade. They identified a reduction in the administrative burden to identify available mental health services. The CM will have dedicated clinical time for in‐depth psychoeducation with birthing people and longitudinal care coordination, a clinician‐identified challenge due to obstetric care demands.

The downstream effects of the CM as a clinic resource may be substantial. Clinicians noted that the pCCM has the potential to reduce moral injury in clinical care by alleviating the distress associated with identifying mental health needs without the capacity to ensure appropriate care. The presence of a dedicated CM to follow a person's mental health trajectory was described as empowering for clinicians, as it offers reassurance that their patients are receiving timely and appropriate care beyond their obstetric management.

## 4 DISCUSSION

This research describes anticipated implementation determinants of pCCMs as reported by key informants. Identified determinants may help inform potential implementation considerations for organizations considering pCCM adoption, such as those presented below.

Because participants identified staff buy‐in and leadership support as important facilitators, these findings suggest that trusted clinician advocates (“Clinician Champions”) may help support engagement during early implementation efforts [37]. Clinician Champions can assist in conceptualization of local adaptations, encouragement of staff engagement, and provision of a trusted channel for bidirectional feedback during implementation.

Identified outer context barriers highlight several areas that may warrant consideration during pre‐implementation planning. First, because clinicians described discomfort with psychotropic prescribing, educational supports and concise prescribing resources may help address this anticipated barrier. Substantial recent growth in accessible educational and clinical support resources for obstetric clinicians include practical treatment guidance, prescribing frameworks, and implementation tools, many of which are summarized in a recent review on preventing maternal morbidity and mortality related to perinatal mental health conditions [38]. Increasing prescribing confidence may facilitate more in‐depth conversations with birthing people about medication use during pregnancy, helping address hesitancy or misconceptions. Participants frequently described the CM role as potentially helpful in supporting patient‐centered discussions and connecting clinicians with educational resources.

Careful selection of the CM may also reduce patient‐centered barriers. As noted by participants, fear of repercussions associated with disclosing perinatal mental health conditions can limit treatment uptake. Populations who experience historic injustice may be less likely to engage with perinatal mental health care [39, 40, 41]. These findings suggest that pre‐implementation discussions with community stakeholders may help inform CM selection and training. Many patients express preferences for concordant care, whether this be racial, ethnic, linguistic, or otherwise, with evidence suggesting enhanced patient‐provider communication, trust, and patient satisfaction with concordant care provision [42, 43, 44]. Further, equity‐informed implementation strategies can be used, such as those described in the Health Equity Implementation Framework [45]. Clinics may consider pre‐implementation listening sessions with community members and a community advisory board to inform program design, as outlined by de Brito et al. [46].

The adoption of the pCCM necessitates changes to existing clinic workflows, an Innovation‐specific barrier. The process of integrating a CM requires process mapping, analysis of existing screening protocols, and team‐based planning to define referral pathways. Participants frequently described workflow integration and role delineation as anticipated barriers to pCCM implementation. These findings suggest that clinics may benefit from structured implementation support during workflow redesign and adaptation. One potential approach described in implementation literature is practice facilitation, which uses interactive problem‐solving and implementation support to assist practice change [47]. Given variation across clinical settings in patient populations, staffing models, and administrative infrastructure, pCCM implementation is inherently context‐dependent, not a one‐size‐fits‐all intervention. Although these findings emerged from a state with relatively robust behavioral health infrastructure, many of the identified determinants may be transferable to lower‐resource settings. Importantly, the themes identified herein may help clinics anticipate barriers related to workforce limitations, clinician discomfort with psychotropic prescribing, and workflow integration prior to implementation. In settings with limited access to specialty mental health care, implementation strategies may rely more heavily on telepsychiatry consultation, community partnerships, and stepped‐care approaches tailored to local resource availability. Thus, rather than prescribing a single implementation model, these findings may support context‐specific adaptation of pCCMs across diverse obstetric practice environments.

Research on CCMs in primary care settings has identified facilitators consistent with our findings, including active involvement from clinic leadership in CCM planning and provision [48, 49], recognition of clear disparities in mental health care access that necessitate integrated behavioral health services, and provider desire for augmented support in managing mental health conditions [50]. Identified barriers also aligned with existing literature, which has demonstrated that social stigma and treatment‐related fears limit CCM engagement, particularly among historically marginalized populations [51, 52].

Results of this qualitative study further build evidence of obstetric‐specific considerations for pCCMs. Prior pCCM implementation research aligns with determinants described herein, including limited comfort among obstetric clinicians with managing perinatal mental health conditions [53] and the need for site‐level workflow adaptations to accommodate obstetric care schedules [25, 54]. These determinants may be particularly salient in obstetric settings because prenatal care involves standardized, high‐frequency encounters occurring within time‐constrained visits, often without embedded behavioral health infrastructure. As a result, workflow integration, role delineation, and clinician comfort managing perinatal mental health conditions may have heightened importance compared to other medical settings. In addition, our findings contribute a more nuanced perspective to patient‐ and provider‐specific themes. For example, the perspective that pCCMs may reduce obstetric clinician burnout extends prior work and may represent an additional motivator for healthcare leadership considering pCCM adoption.

## 5 LIMITATIONS

It is important to acknowledge that these findings reflect perspectives from Rhode Island, a relatively small state with a population of just over 1 million people,  broad insurance access through Medicaid expansion, and a comparatively robust mental health care infrastructure. While this is noted as a potential facilitator of COMPASS+ implementation, applications of similar models in other settings should view this determinant according to the unique care landscape, with particular pre‐implementation attention to insurance coverage and referral networks. Other notable limitations of this study include the time point of data collection in the pre‐implementation phase; therefore, results cannot be interpreted alongside implementation outcomes. In addition, all interviews were conducted by a single interviewer. While this decision was intentionally selected to maximize consistency and standardize data collection, it may have unintentionally introduced interviewer bias. Finally, because the EPIS framework guided analysis from the outset, it is possible that the framework unduly influenced the interpretation of the data. This risk was mitigated via thematic debriefing with two separate members of the research team experienced in many different qualitative implementation frameworks.

## 6 CONCLUSIONS

In light of the significant public health impact of perinatal mental health conditions, innovations in care delivery, such as the pCCM, represent a promising approach to addressing unmet mental health needs in obstetric settings. Participants in this qualitative pre‐implementation evaluation described the pCCM as having potential for integration within obstetric settings given multiple touchpoints within the prenatal care structure, patient trust in obstetric clinicians, and the need for more coordinated behavioral health support. However, participants also identified important barriers related to workflow integration, workforce limitations, clinician training, and sustainability, highlighting the need for context‐specific adaptation during implementation planning. By attending to inner‐, outer‐, and innovation‐related factors, health systems can more effectively bridge the gap between evidence and routine practice, supporting broader dissemination of this evidence‐informed model of care.

## CONFLICT OF INTEREST STATEMENT

The authors declare no conflicts of interest.

## ETHICS STATEMENT

We confirm that all research adhered to safety, privacy, and integrity standards as outlined by the Institutional Review Board at Women and Infants Hospital. This research was deemed exempt given that the proposed activity does not involve human subjects, as defined by DHHS and FDA regulations.

## Supporting information



Supporting Information

## Data Availability

The data that support the findings of this study are available from the corresponding author upon reasonable request.
