# Supplementary material for: Scaling the collaborative care model in obstetrics: Multilevel determinants to guide implementation
Source: Pregnancy (Hoboken). 2026 Jul 7;2(4):e70356. doi: 10.1002/pmf2.70356 (PMC13344441; doi:10.1002/pmf2.70356)
Supplement: Supplementary file 1 [file PMF2-2-e70356-s001.docx]

Interview Guide: COMPASS+

**Purpose**: The purpose of these in-depth qualitative interviews is to explore obstetric care provider’s and clinic support staff’s prospective on barriers and facilitators to the care cascade surrounding perinatal mental health conditions. We are seeking to understand these prospectives to inform delivery of the COMPASS+ intervention and which specific factors affect the care of perinatal mental health conditions in existing medical infrastructure and related to the proposed expansion of behavioral health services.

**Procedures**: Participants will be selected on a voluntary basis from the COMPASS+ Project clinic cohorts before the pre-implementation period. Interviews will be semi-structured, last approximately 45 minutes. Interviews will be recorded for future analysis and will be stored in a password-protected folder on a secure sever. PHI will be redacted during coding and publication.

**Interview Guide**:

I. Introduction
   a. **Purpose**: To introduce the facilitator, describe the purpose of the interview, and asses understanding of the COMPASS+ Program. Before interview begins, there will be time for questions and a review on confidentiality.
II. Obstetric provider roles
    a. **Purpose**: To explore perspectives on screening and treating perinatal mental health conditions.
    b. **Provider-specific considerations**:
       i. Tell me about training you have had to help you address mental health conditions in your patients.
       ii. What are resources you turn to when managing and treating mental health conditions in your patients?
       iii. Do you feel comfortable prescribing medications to manage perinatal mental health conditions? For what conditions? For how long?
       iv. Is there anything that limits your ability to effectively treat perinatal mental health conditions?
    c. **Current landscape**:
       i. Walk me through a scenario when a patient has an elevated screen for a mental health disorder. What are next steps?
       ii. What are screening/diagnosis/management barriers?
       iii. How do you balance different demands during a patient visit?
III. Compass+ Expansion
     a. **Purpose**: To discuss the proposed expansion of COMPASS+ with clinic members including a review of care manager’s proposed role.
     b. **Care Manager**
i. Who do you think the care manager should be? What aspects of the population you serve informs this?
   a. Licensing and Training?
   b. Characteristics? (bilingual, bicultural)
ii. How do you envision a care manager fitting into your existing workflow?
iii. What specific changes to your workflow and clinic in general will need to be made to integrate a care manager?
iv. How would you define success within the context of the COMPASS+ study?
    a. Screening rates? Interactions with patients?
    c. **Adoption**
       i. Do you feel prepared to adopt COMPASS+ in your clinic?
       ii. What are you excited about in the expansion of behavioral health services in your clinic?
       iii. What do you anticipate as the biggest barriers with COMPASS+ in your clinic?
       i. Who do you think this program will benefit most and why?
       ii. What do you think your clinic will get out of participating in COMPASS+?
IV. Conclusion
